# Control of Peach Brown Rot Disease Produced by *Monilinia fructicola* and *Monilinia laxa* Using Benzylidene-Cycloalkanones

**DOI:** 10.3390/jof10090609

**Published:** 2024-08-27

**Authors:** Alejandro Madrid, Valentina Silva, Constanza Reyes, Enrique Werner, Ximena Besoain, Iván Montenegro, Evelyn Muñoz, Katy Díaz

**Affiliations:** 1 Laboratorio de Productos Naturales y Síntesis Orgánica (LPNSO), Departamento de Ciencias y Geografía, Facultad de Ciencias Naturales y Exactas, Universidad de Playa Ancha, Avda. Leopoldo Carvallo 270, Playa Ancha, Valparaíso 2340000, Chile; silvapedrerosv@gmail.com (V.S.); constanza.reyesv@alumnos.uv.cl (C.R.); evdmunoz@gmail.com (E.M.); 2 Departamento de Ciencias Básicas, Campus Fernando May, Universidad del Bío-Bío, Avda. Andrés Bello 720, Casilla 447, Chillán 3780000, Chile; ewerner@ubiobio.cl; 3 Escuela de Agronomía, Pontificia Universidad Católica de Valparaíso, San Francisco s/n La Palma, Quillota 2260000, Chile; ximena.besoain@pucv.cl; 4 Center of Interdisciplinary Biomedical and Engineering Research for Health (MEDING), Escuela de Obstetricia y Puericultura, Facultad de Medicina, Universidad de Valparaíso, Angamos 655, Reñaca 2520000, Chile; ivan.montenegro@uv.cl; 5 Laboratorio de Pruebas Biológicas, Departamento de Química, Universidad Técnica Federico Santa María, Av. España N1680, Valparaíso 2340000, Chile

**Keywords:** dihydrocarvone, *Monilinia* spp., postharvest fruits, nectarines

## Abstract

Fruit rots caused by filamentous fungi such as *Monilinia fructicola* and *Monilinia laxa* have a strong impact on crop yield and fruit commercialization, especially as they affect a wide variety of stone fruits. The antifungal efficacy of benzylidene-cycloalkanones has been previously described in in vitro assays against *M. fructicola*; so, this study aims to evaluate the in vivo inhibitory potential of these hybrids on fruits that have been inoculated with *M. fructicola,* and use molecular docking to visualize the main interactions of these molecules in the active site of the enzyme succinate dehydrogenase (SDH). The results indicate that compound **C** achieves the highest inhibition of both *Monilinia* species (15.7–31.4 µg/mL), spore germination in vitro (<10 µg/mL), and has promising results in vivo, without causing phytotoxicity in fruits. The results from molecular docking suggest that hydroxyl groups play a crucial role in enhancing the binding of compound **C** to SDH and contribute to the formation of hydrogen bonds with amino acid residues on the enzyme active site.

## 1. Introduction

Fungi diseases that affect fruit cause devastating damage to commercial crop plants pre- and post-harvest, resulting in large economic losses. Among those causing these are the filamentous fungi *Monilinia laxa* (Aderhold and Ruhland) Honey and *Monilinia fructicola* (G. Winter) Honey [1]. Its main hosts are stone fruits such as cherries, peaches, and nectarines, among others, in which it causes brown rot. The onset of the disease is characterized by the appearance of a light brown, soft and watery spot that develops rapidly after harvesting, especially if the fruit is wounded [2], reaching losses of up to 10% of post-harvest productivity worldwide [3]. The genus *Monilinia* belongs to the family Sclerotiniaceae, of the phylum Ascomycota, and its distinctive feature is that the fruiting body originates from pseudosclerotia formed in mummified fruits in soil or their remains, from which spores are produced [4]. Disease, mycelium development and sporulation find their ideal conditions at a relative humidity of more than 80% and at a temperature of approximately 25 °C. *M. laxa* is most prevalent worldwide in orchards [5]; however, *M. fructicola* is more aggressive and has been considered a quarantine pest in several stone-fruit-producing countries [6] and is less sensitive to fungicides used for its control [4], especially to Methyl Benzimidazole Carbamates (MBCs); its resistance mechanism is associated with mutations in the binding site of the β2-tubulin proteins, which are part of the mechanism of action of these antimicrobials [7,8].

**Figure 1:**
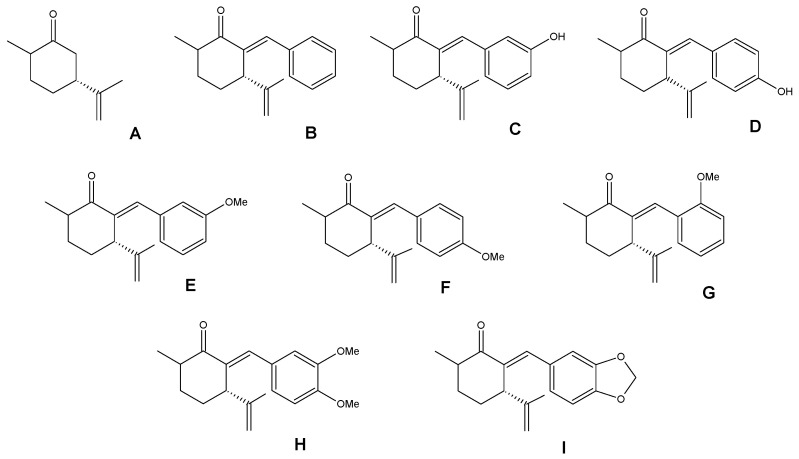
Dihydrocarvone (**A**) and its previously reported benzylidene-cycloalkanones (**B**–**I**).

Considering the challenges of controlling *Monilinia* spp. and its catastrophic effect on fruit productivity, strategies have been sought, such as avoiding post-harvest conditions that favor the disease, the use of biological control agents [9] and the use of natural bioactive compounds [10]. Regarding the latter group, the metabolites used are those produced by plants in interaction with the environment and associated with their own defense mechanisms so that they can have antifungal activity [3]. Among these are molecules with confirmed antifungal capacity, such as terpenic ketones, one of which is dihydrocarvone, a major compound in the essential oil of *Poiretia latifolia* flowers [11]. Díaz et al. 2021, in a previous publication, demonstrated the antifungal activity of dihydrocarvone hybrids, namely, benzylidene-cycloalkanones (shown in Figure 1), which had remarkable efficacy and low toxicity in *Artemia salina* models, which is significant considering that their application will be on edible fruits [12]. Therefore, we will extend this study to determine the effect of the dihydrocarvone (**A**) and its nine benzylidene-cycloalkanones (**B**–**I**) on conidial germination and inhibitory effect on nectarine fruits inoculated with *M. fructicola*; additionally, we will visualize the main interactions with the active site of the enzyme succinate dehydrogenase (SDH) by molecular docking studies.

## 2. Materials and Methods

### 2.1. General Chemistry

Dihydrocarvone (**A**), reagents and chemicals were obtained commercially from Sigma-Aldrich Co. (St. Louis, MO, USA) and AK Scientific Inc. (30023 Ahern Ave, Union City, CA, USA) and were used unpurified. The benzylidene-cycloalkanones (**B**–**I**) were available in the laboratory due to previous research and their NMR data can be found in the Supplementary Materials.

### 2.2. Assessment of Antifungal Capacity

#### 2.2.1. Fungal Isolate and Growing Conditions

The isolates of *Monilinia fructicola* and *M. laxa* used in these experiments were provided by the collection of the Mycology Unit, Servicio Agrícola y Ganadero (SAG), Chile. Cultures of the isolate were grown and incubated on potato dextrose agar (PDA) medium in 90 mm diameter Petri dishes under aseptic conditions at 23 °C for 7 days, and subsequently stored at 4 °C. The spore suspension applied in the in vitro studies was obtained from a 7–10-day growth culture in Petri dishes, which were filtered through a sterile muslin cloth [13]. Spore concentration was adjusted through serial dilution in sterile distilled water.

#### 2.2.2. Effect of the Benzylidene-Cycloalkanones Compounds on the Mycelial Growth on *Monilinia laxa*

Determination of mycelial growth inhibition of the samples was performed by measuring the radius of growth. The PDA plates contained the compounds (dihydrocarvone **A** and benzylidene-cycloalkanones **B**–**I**) at concentrations of 10, 25, 50, 150 and 250 µg/mL previously dissolved in ethanol and water. The percentage of inhibition was determined according to standard methods [12]. PDA 1% ethanol medium was used as a negative control, while a commercial fungicide, Mystic^®^ 520 SC (BAYER AG, Leverkusen, Germany), was used as a positive control; also, a commercial organic fungicide based on grapefruit extracts, BC-1000^®^ Dust (CHEMIE Research & Manufacturing, Casselberry, FL, USA), was used. The results are expressed as the average effective concentration (EC_50_), that is, the concentration at which mycelial growth was reduced by 50%. This value was determined by regression of the values of the percentage inhibition of the radial growth versus the concentration values of the compound using Origin ProV. 8 software (OriginLab Corporation, Northampton, MA, USA). Treatments were performed in triplicate.

#### 2.2.3. Effect of the Compounds on Conidial Germination of *Monilinia fructicola*

To evaluate the effect of the compounds (**B**–**I**) on conidial germination following the protocol previously described by Pereira et al., 2017, with modifications [14], *M. fructicola* 6-day-old colonies grown on PDA at 22 °C were used to prepare spore suspensions at a concentration of 1 × 10^5^ conidia/mL. A 40 µL aliquot of the conidial suspension was spread on the media with different treatments containing compounds at the final concentrations of 10, 25, 50, 50, 150, and 250 µg/mL. Sterile distilled water was used as a negative control. The plates containing the dispersed conidia on media were stored at 22 °C for 9 to 12 h in the dark. Approximately 100 spores were randomly measured under a microscope to determine their percentage of germination (PIG); each treatment consisted of three replicates. Conidia were considered germinated if they produced germ tubes at least twice their width. The results are expressed as the average effective concentration (EC_50_), that is, the concentration at which inhibition conidial was reduced by 50%.

#### 2.2.4. In Vivo Antifungal Effects of Hybrids Based on Dihydrocarvone (**B**, **C** and **G**) on Inoculated Nectarines Fruits

The assay was performed following the methodology described by Brito et al., 2021, with slight modifications [13] on commercially obtained nectarines (*Prunus persica* var. *nucipersica*). The selected fruits were healthy. Their surface was sterilized by a 10 min immersion in a 15% sodium hypochlorite solution, and then washed and dried with sterile absorbent paper to be inoculated with *M. fructicola*. Artificial inoculation was carried out at room temperature by puncturing the fruit with a sterile needle at four separate points, and 100 µL of a suspension containing 1 × 10^6^ CFU/mL of fungal spores was applied to each puncture. One day before inoculation, the fruits were sprayed with the selected treatments, **B**, **C** and **G,** at a concentration of 250 µg/mL; the selected compounds were defined according to their in vitro inhibition concentrations of low, medium and high efficacy. As a negative control, wounded fruits sprayed with sterile distilled water were used, as well as non-inoculated fruits that were sprayed to observe if there was phytotoxicity of the treatments. Each experiment consisted of three replicates and each replica had four nectarines. Organic fungicide BC1000^®^ Dust was used as a positive control at the same compound concentrations.

**Figure 2:**
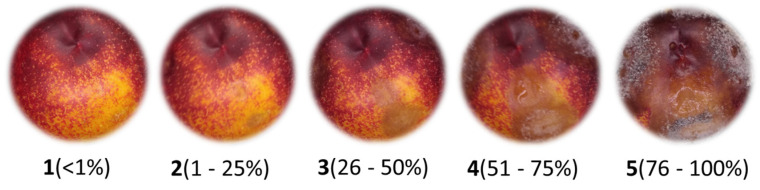
Visual scale (1–5) of symptoms caused by *M. fructicola* to quantify the incidence and severity of the disease on nectarines at 5 days post-inoculation: **1** = no sign of disease (not infected); **2** = 1–25% lesion visible but no sporulation (mild infection); **3** = 26–50% sporulating area on lesion smaller than a quarter of the fruit (moderate infection); **4** = 51–75% sporulating area larger than a quarter of the fruit, but less than half of the fruit; (strong infection); **5** = 76–100% sporulating area larger than half of the fruit (severe infection).

They were kept at room temperature (25 °C), under light, in a humid chamber with a relative humidity of about 95% for 5 days in dark. After 4 days of incubation, the percent disease incidence (PIC) was calculated as PIC = (number of infected lesions)/(number of lesions evaluated) × 100. The disease severity index (DSEI) was calculated using the Townsend–Heuberger equation [15]. This equation uses a visual symptom scale described in Figure 2 [16].

The presence of the pathogen was verified by re-isolating it from the edge of the lesions; a slice (1.5 cm) was cut and the tissue was placed in Petri dishes with PDA (applying Koch’s postulates), which were inspected and morphologically identified using a Leica EZ4HD stereo microscope with camera software and DM500 microscope (Leica Microsystems, Wetzlar, Germany).

### 2.3. Statistical Analysis

Statistical analysis was performed with the program InfoStat v.2020, (Universidad de Cordoba, Córdoba, Argentina) and using the multi-comparison function with a least significance difference test (LSD) with a *p* ≤ 0.05 of significance.

### 2.4. Molecular Docking and Prediction of the Physicochemical Properties

The construction of three-dimensional models of the ligands was performed using Avogadro 1.2.0n software. The geometry of the ligands was optimized, and their energy was minimized using the MMFF94 force field. Images were created using the Discovery-Studio visualize free version. The crystallized structure of succinate dehydrogenase (SDH PDB ID: 2FBW, 2.06 Å resolution) [17] was obtained from the Protein Data Bank (http://www.resb.org/pdb). Molecular docking of SDH and the selected ligands was carried out with the AutoDock4 package using the Lamarckian genetic algorithm [18] and assuming rigid ligands in the macromolecule and full flexibility for the inhibitors. The search parameters were 50 runs and a maximum number of 25,000,000 evaluations for each ligand. The RMSD threshold for multiple clustering was set at <0.5 Å. The ligand crystallized in the enzyme 2-methyl-n-phenyl-5,6-dihydro-1,4-oxathiine-3-carboxamide (CBE) was used as a reference point for docking. The result was analyzed by ranked cluster and binding energy ΔG, where the lowest energy and most populated cluster was selected as the best protein–ligand complex for further analysis. To check the docking accuracy, the co-crystallized ligand was docked again under the same conditions and an RMSD of 2.49 Å was obtained. All experiments were performed at physiological pH. Prediction of the physicochemical properties of the compounds was compiled using Swiss ADME software (http://www.swissadme.ch/index.php).

## 3. Results and Discussion

### 3.1. Antifungal Activity of Dihydrocarvone and Its Hybrid Derivatives

The antifungal activity of the compounds (Table 1) determined from the mycelial growth inhibition of *Monilinia* spp. showed that *M. laxa* was the most sensitive pathogen to the treatments, achieving mean growth inhibition at concentrations within the range of 8.38 to 55.97 µg/mL, with the exception of sample **A**, corresponding to the starting compound which was inactive for both species, suggesting that the modifications made to obtain the hybrids improved their antifungal activity. Structure–activity relationship studies of synthetic and natural product derivates relate the presence of hydroxyl groups to improved solubility and pharmacokinetic properties, including beneficial effects on the formation of indirect hydrogen bonds through water molecules to the active site [19]. Among the molecules synthesized, **C** stands out for the presence of a hydroxyl group and its high antifungal capacity in *M. fructicola* compared to the rest of the treatments.

It is worth mentioning that it is evident that samples **B** and **D** are inactive in *M. fructicola*; however, they have an EC_50_ of 8.38 and 27.30 µg/mL in *M. laxa*, respectively, and this difference in sensitivity has also been described by other authors [3]. Studies of other monoterpenoids of natural origin also attribute activity to them in *M. fructicola* [20] and have shown that monoterpenes such as thymol are effective against it, where the compound crystallizes on the surface of the conidia, hyphae and cell wall of the fungus, being absent in the cytoplasm [21]. Likewise, the antifungal capacity of monoterpenes was also attributed to the presence of a phenolic hydroxyl group [22]. These discrepancies in pathogen–compound interaction could be attributed to culture specificity, presence or absence of specific receptors and resistance to death mechanisms.

Regarding the conidia of this species, which can be found in mummified fruits, where the infection can start, they are dispersed by water or wind, and subsequently infect the different tissues of the plant. These conidia are able to germinate at an optimum temperature between 15 and 30 °C; however, they can germinate even in a range of 0 to 35 °C [3]. Although brown rot is a disease that appears more frequently post-harvest at the points of contact between the fruits [23], infection of the fruit usually occurs while they are still on the tree, remaining latent until harvest; therefore, a treatment for this disease must not only control the development of *Monilinia* spp. but also be able to inhibit the conidia present on the surface of the fruit. Most of the treatments tested in this study were highly effective in inhibiting conidia germination (<10 µg/mL), with the exception of precursor **A** and hybrid **H**.

Table 1 also shows the lipophilicity of the compounds expressed as Log P, and these physicochemical parameters were obtained from the SwissADME platform. Compound **A** is the one that experimentally shows the lowest mycelial inhibition capacity of the two *Monilinia* species and is the compound with the lowest lipophilicity. The rest of the compounds have Log P values greater than 3, which indicates that they are more lipophilic compounds with moderate water solubility. For a molecule to exert biological action on a cell, it must meet certain physical and chemical requirements, including the ability to diffuse through the cell membrane. The speed at which diffusion occurs depends largely on the lipid solubility of the molecule. Since the cell membrane is lipid in nature, liposoluble molecules diffuse more quickly through it [24]. The balance between lipophilicity and hydrophilicity is of great importance in biological processes, since if the lipophilicity of the compounds is too high, the desired effects are not achieved either; for example, compound **B** shows a Log P value of 4.02, being the most lipophilic compound of those evaluated. Additional physicochemical properties of the compounds such as the number of rotatable bonds, H-bond acceptors, H-bond donors, molar refractivity and topological polar surface area can be found in Supplementary Materials.

To summarize, the derivatives of dihydrocarvone hybrids described by Díaz et al. [12] are active on *Monilinia* spp., especially on *M. laxa*, two compounds (**B** and **I**) proved to be more active than the positive controls in inhibiting mycelial growth and germination of conidia of this species. However, due to the relevance of *M. fructicola* for its devastating effect on commercial crops, difficult control and resistance, compound **C** was selected as the most effective candidate in the control of *Monilinia* spp. for the purposes of this study, as it was able to inhibit *M. fructicola* at a concentration of 15.7 µg/mL, *M. laxa* at a concentration of 31.46 µg/mL and to achieve an outstanding inhibition on conidia.

### 3.2. In Vivo Antifungal Effects of Hybrids Based on Dihydrocarvone (**B**, **C** and **G**) on Inoculated Nectarine Fruits

**Figure 3:**
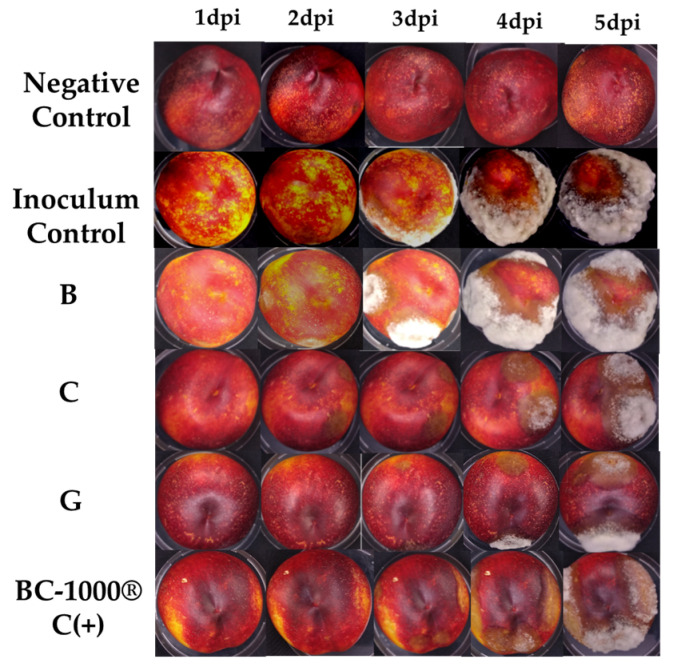
Effects of compounds **B**, **C** and **G** at 250 µg/mL controlling the development of *M. fructicola* in artificially inoculated nectarines for five days post inoculation (dpi)**.** Negative Control: without inoculum; Inoculum Control: with inoculum of *M. fructicola*; **B**: compound of low antifungal activity in vitro; **C**: compound with antifungal activity similar at positive control in vitro; **G**: compound of intermediate antifungal activity; BC-1000^®^ Dust.

To determine the effects of the hybrids on fruit, commercially obtained nectarines were chosen to be inoculated with *M. fructicola* (Figure 3); before this, treatments were applied with three derivatives that were selected according to the results obtained in the in vitro tests. Compound **C** presented activity similar to the positive control, **G** presented intermediate activity, and **B** was proven to be a compound of low antifungal activity, thus determining a correlation with the in vivo results. Figure 3 shows the severity of damage in fruits inoculated with the pathogen treated with the different treatments, showing the percentage of disease incidence at 1–5 dpi (Table 2). This trial demonstrates the protective effect that the compounds had on disease development. As expected, according to the results obtained in the previous stage, fruits treated with compound **B** had a higher disease development. Likewise, **C** and **G** had an excellent inhibitory effect on disease incidence up to the fourth day after inoculation, showing the first symptoms on the fifth day with lower incidence in fruits treated with hybrid **C**. On the other hand, the positive control BC-1000^®^ Dust showed symptoms on the fourth day with low incidence; however, on the fifth day, disease incidence increased to values close to 100%.

**Figure 4:**
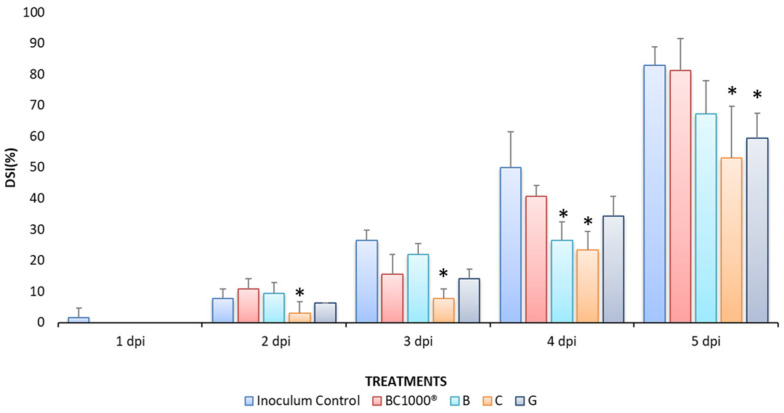
Disease severity index (%DSI) on nectarine fruits measured for five days post-inoculation (dpi) with *Monilinia fructicola*. Mean with asterisk (*) denotes a statistically significant difference between BC-1000^®^ Dust treatment and compounds according to analysis of variance (ANOVA, *p* < 0.05) and comparison of the groups using Fisher’s LSD. Data are expressed as means ± SD.

In general, the results obtained in the in vivo trials were consistent with the results obtained in vitro in terms of the structure–activity relationship. The effect on disease inhibition was enhanced when the molecule had a hydroxyl group at the C-3 position compared to when there was a methoxyl group at the C-2 position of the molecule, and decreased disease severity by 6–12%. In contrast, the disease did not decrease when the molecule did not have functional groups attached to the aromatic ring, which accelerated the infection during the bioassay, as in the case of compound **B** (Figure 4). The disease severity index (DSI) was assessed using a visual scale and calculated with the Townsend–Heuberger equation [15]. The results are presented in Figure 4 as the percentage of damaged fruit being the highest result for the control treatment that was inoculated with the pathogen, and the positive control, which, prior to inoculation, was sprayed with a commercial organic pesticide used in different vegetable crops (BC-1000^®^), unlike all other treatments that reduce the disease index, and could have maintained the control of the pathogen if it had been inoculated again with these compounds. However, the fruits only received one application. It is expected in the future to conduct new experiments that include a second one to reduce the disease index.

It has been previously described that *Monilinia* spp. are intercellularly lodged [3] and, upon infection of fruits, respond with the accumulation of proteins [25], and activation of the jasmonic acid and ethylene pathways, in addition to phenylpropanoid metabolism, among other responses. In the pathogen, the activation of genes related to transporters, phospholipases, oxidoreductases, and proteases occurs [26], in addition to the secretion of other enzymes characteristic of necrotrophic fungi such as *Monilinia* spp. The cascade of molecules exhibited by the pathogen and fruits in their interaction [27] is a factor to consider because it could interfere with the mechanism of action of the proposed hybrids, which is why it is necessary to test their activity in in vivo models. In addition, the use of in vivo models allows us to evaluate possible phytotoxicity reactions of the compound on the fruits; the compounds evaluated did not cause changes in the appearance of the fruits or phytotoxicity.

Different varieties of stone fruit can be affected by *Monilinia* spp., and among these are cherries, plums, peaches, etc.; however, nectarines were chosen for this study since their size allows for the visualization of the effects of the treatments on the disease. The results could be extrapolated to other fruits, considering that the peel of fruits in general is composed of biopolymer cellulose and lignin and of usual compounds of the plant cell wall [28]. Therefore, these dihydrocarvone hybrids could be used as potential organic fungicides in more than one stone fruit crop.

### 3.3. Molecular Docking of Benzylidene-Cycloalkanones **B**, **G** and **C**

Succinate dehydrogenase, also known as succinate coenzyme Q reductase or mitochondrial complex II, (SDH) is a protein bound to the inner mitochondrial membrane. This protein, present in aerobic cells, participates in the Krebs cycle as well as in the electron transport chain. This enzyme is made up of four protein subunits, subunits A and B of a hydrophilic nature, while subunits C and D of a hydrophobic nature are attached to the mitochondrial membrane [29]. Various investigations have shown that SDH is an ideal objective and one of the preferred targets for the development of new fungicides [30,31,32]. SDH-inhibiting pesticides are an important class of agricultural fungicides with the advantages of high efficiency and have also been shown to act as bactericides on a broad bacterial spectrum [33]. The literature shows that there is a wide structural diversity of potential SDH inhibitors. A previous work reported by Soto et al. [34] indicates that the antifungal activity on *Botrytis cinerea* caused by hydrated geranylated phenols could be through the inhibition of the SDH enzyme. 

The molecular docking of the dihydrocarvone derivatives was performed on the crystal structure of SDH obtained from the PDB Database (PDB: 2FBW). 2-methyl-n-phenyl-5,6-dihydro-1,4-oxathiine-3-carboxamide was used as a reference ligand, which corresponds to the ligand crystallized in the enzyme. The compounds evaluated in vivo were chosen to measure the activity on SDH. The results are shown in Table 3, which indicates the binding energy (Kcal/mol) of compounds **B**, **G** and **C,** which were considered in order of increasing activity, with compound **C** showing the best activity of the set of molecules evaluated.

**Figure 5:**
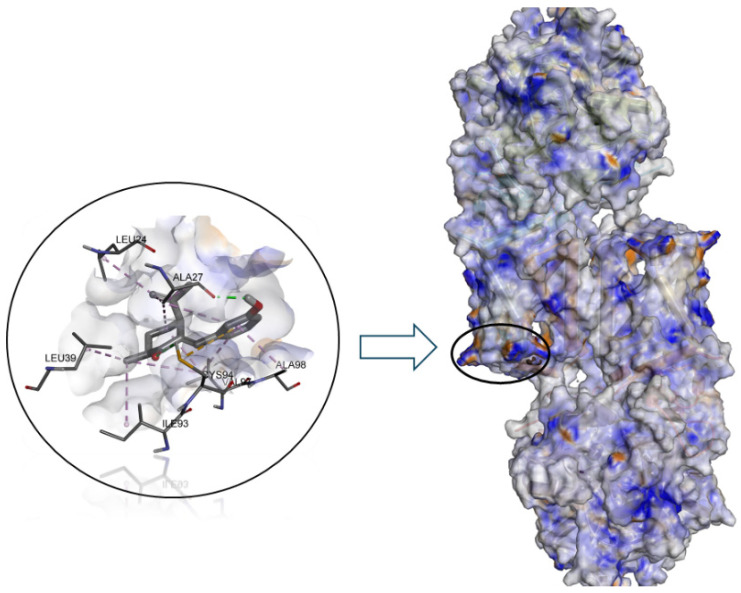
Predicted binding mode of compound **C** into the allosteric site of enzyme SDH (PDB 2FBW).

We can observe that the binding energy values were −5.3 for **B**, −6.6 for **G** and −7.1 for **C**. As can be seen in Figure 5, the binding site of all dihydrocarvone derivatives to SDH would be in the D subunit of this enzyme. It is worth noting that there is a high correlation between the results obtained theoretically and experimentally, with the R2 value for *M. fructicola* being 0.90 and for *M. laxa* being 0.94.

**Figure 6:**
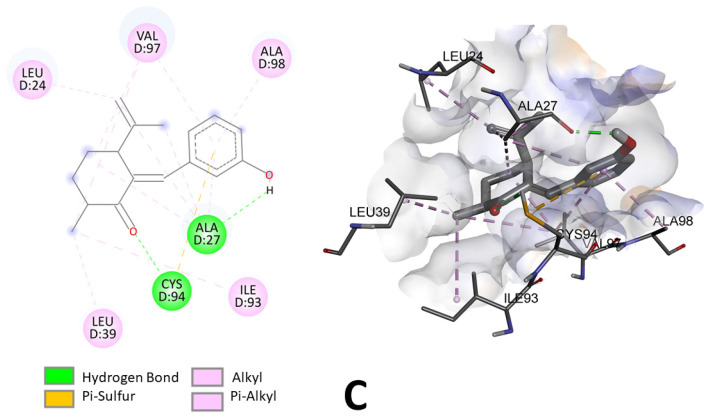
Two-dimensional and three-dimensional predicted binding mode of compound **C** into the allosteric site of enzyme SDH (PDB 2FBW).

Compound **C** was the one that had the greatest effect on the inhibition of mycelial growth of *Monilinia* spp. This compound acts as a ligand binding to the amino acid residues leucine-24, valine-97, alanine-98, alanine-27, cysteine-94 and leucine-39. As can be seen in Figure 6, two hydrogen bonds would be formed between compound **C** and SDH, one at 2.83 Å distance between the carbonyl group of the compound and the cysteine-94 residue and a second hydrogen bond at 2.12 Å between the hydroxyl group and the alanine-27 residue. This same compound shows a π–sulfur-type bond between the phenolic ring (5.72 Å) and the cysteine-94 residue. Several alkyl and π–alkyl interactions can also be observed, which are considered to be lower-intensity interactions compared to hydrogen and sulfide bonds [35]. 

**Figure 7:**
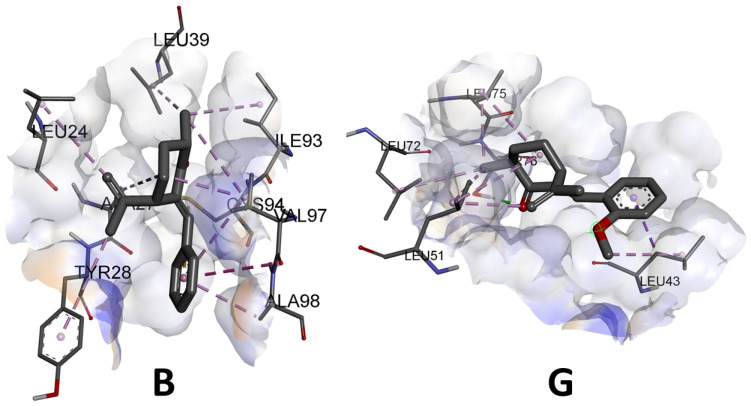
Three-dimensional predicted binding mode of compound **B** and **G** into the allosteric site of enzyme SDH (PDB 2FBW).

As previously mentioned, the activity of the compounds on SDH is related to the number of hydrogen bonds that can be generated between the ligands and the amino acid residues of the enzyme [19]. Most of the benzylidene-cycloalkanone derivatives form hydrogen bonds and it should be noted that the most active compound (**C**) forms two hydrogen bonds with the enzyme, whereas, as can be seen in Figure 7, the compound evaluated in vivo with the lowest activity (**B**) would not generate this type of interaction with the enzyme; rather, weaker alkyl interactions predominate. Compound **G** forms a single hydrogen bond. It should also be noted that the most active compound generated a sulfur bond with the enzyme, which would explain the greater stability of the complexes obtained.

## 4. Conclusions

The benzylidene-cycloalkanones tested confirmed their antifungal potential against *M. fructicola* and *M. laxa* strains, demonstrating the effectiveness of pathogen growth inhibition in vitro and disease control in vivo. The structure of compound **C** is the most decisive in inhibiting the growth of *M. fructicola*, owing to the presence of a hydroxyl group on carbon 3 of the aromatic ring, which allows it to bind to the active site of the SDH enzyme.

A molecular docking study shows that binding energies of these compounds to the allosteric binding site of SDH are −7.1 kcal mol^−1^ similar to CBE. The results suggest that the experimental antifungal activity correlates with the number of H-bonds that can be formed in the binding site.

For future studies, the application of new synthesis methodologies could be explored to improve their performance and reduce reaction times due to their valuable activity. For this reason, it is also necessary to investigate the mechanism of action through which the hybrids cause the inhibition of the pathogen. Considering that the potential application of these compounds would be in the agricultural industry, technologies that could be used as a vehicle for their application could be evaluated with the use of nanoemulsions loaded with the active compounds, a technology that is valuable in this industry due to its advantages in cost and safety. On the other hand, it would also be interesting to explore if its application results in changes in the taste of fruits, or if it represents any risk of oral toxicity.

## Figures and Tables

**Table 1:** Lipophilicity and EC_50_ values of dihydrocarvone (**A**) and its derivatives (**B**–**I**) calculated for inhibition of mycelial growth of *Monilinia* spp. in vitro and inhibition conidial germination of *M. fructicola*.

Compound	EC_50_ (µg/mL) ± SD ^a^			Lipophilicity Log *P*
	*M. fructicola* ^d^	*M. laxa*	Conidia *M. fructicola* ^e^	
**A**	>250	>250	>250	2.51
**B**	>250	8.38 ± 0.68	<10	4.02
**C**	15.7 ± 1.33	31.46 ± 2.82	<10	3.61
**D**	>250	27.30 ± 0.04	10	3.60
**E**	39.64 ± 1.13	42.49 ± 1.04	<10	4.01
**F**	23.17 ± 1.22	10.88 ± 2.82	<10	4.01
**G**	58.03 ± 0.74	55.97 ± 0.98	<10	3.98
**H**	47.79 ± 0.88	7.57 ± 0.48	>250	3.96
**I**	18.13 ± 0.0	5.06 ± 1.05	<10	3.84
**Control A ^b^**	9.19 ± 0.0	10.56 ± 0.0	<10	-
**Control B ^c^**	10.55 ± 1.74	13.64 ± 1.38	110 ± 1.09	-

^a^ EC_50_: concentration causing 50% mycelial growth inhibition + SD, standard deviation; values are expressed as the mean of three experiments; ^b^ Control A: Mystic^®^ 520 SC; ^c^ Control B: BC-1000 ^®^ Dust; ^d^ data from Díaz et al., 2021 [12]; ^e^ conidial germination.

**Table 2:** Effect of different treatments applied in vivo on nectarine fruits on the incidence of peach brown rot disease over five days post-inoculation (dpi).

Disease Incidence Percentage of Peach Brown Rot Disease (%) ± SD [*]					
Treatments	1 dpi	2 dpi	3 dpi	4 dpi	5 dpi
Negative Control	0 ± 0.0 ^b^	0 ± 0.0 ^e^	0 ± 0.0 ^e^	0 ± 0.0 ^c^	0± 0.0 ^b^
Inoculum Control	6 ± 3.1 ^a^	31 ± 13 ^c^	100 ± 0.0 ^d^	100 ± 0.0 ^a^	100 ± 0.0 ^a^
BC1000^®^ Dust	0 ± 0.0 ^b^	44 ± 10 ^d^	69 ± 13 ^b^	100 ± 0.0 ^a^	100 ± 0.0 ^a^
**B**	0 ± 0.0 ^b^	38 ± 14 ^c^	88 ± 14 ^c^	88 ± 14 ^b^	100 ± 0.0 ^a^
**C**	0 ± 0.0 ^b^	13 ± 3.0 ^a^	31 ± 13 ^a^	88 ± 10 ^b^	94 ± 13 ^a^
**G**	0 ± 0.0 ^b^	25 ± 0.0 ^b^	56 ± 10 ^b^	100 ± 0.0 ^a^	100 ± 0.0 ^a^

[*] Same letters in superscript do not present significant differences (*p* ≤ 0.05); **B**: compound of low antifungal activity in vitro; **C**: compound with antifungal activity similar at positive control in vitro; **G**: compound of intermediate antifungal activity.

**Table 3:** Binding energies obtained from molecular docking studies on SDH. CBE: 2-methyl-n-phenyl-5,6-dihydro-1,4-oxathiine-3-carboxamide, ligand crystalized in SDH (2FBW).

Compound	Binding Energy (Kcal/mol)
**B**	−5.3
**C**	−7.1
**G**	−6.6
CBE	−7.2

**B**: compound of low antifungal activity in vitro; **C**: compound with antifungal activity similar at positive control in vitro; **G**: compound of intermediate antifungal activity.

## Data Availability

The original contributions presented in this study are included in this article/Supplementary Materials; further inquiries can be directed to the corresponding authors.

## Supplementary Materials

The following supporting information can be downloaded at: https://www.mdpi.com/article/10.3390/jof10090609/s1. NMR data and additional physicochemical properties (number of rotatable bonds, H-bond acceptors; H-bond donors, molar refractivity and topological polar surface area) for dihydrocarvone derivatives are available in the Supplementary Materials.
