# Supplementary material for: Control of Peach Brown Rot Disease Produced by Monilinia fructicola and Monilinia laxa Using Benzylidene-Cycloalkanones
Source: J Fungi (Basel). 2024 Aug 27;10(9):609. doi: 10.3390/jof10090609 (PMC11432840; doi:10.3390/jof10090609)
Supplement: Supplementary file 1 [file jof-10-00609-s001.zip › jof-3171136-supplementary.pdf]

SUPPORTING INFORMATION

Control of Peach Brown Rot Disease Produced by *Monilinia fructicola* and *Monilinia laxa* Using Benzylidene-Cycloalkanones

Alejandro Madrid [1,*], Valentina Silva [1], Constanza Reyes [1], Enrique Werner [2], Ximena Besoain [3], Iván Montenegro [4], Evelyn Muñoz [1] and Katy Díaz [5,*]

[1] Laboratorio de Productos Naturales y Síntesis Orgánica (LPNSO), Departamento de Ciencias y Geografía, Facultad de Ciencias Naturales y Exactas, Universidad de Playa Ancha, Avda. Leopoldo Carvallo 270,
Playa Ancha, Valparaíso 2340000, Chile; silvapedrerosv@gmail.com (V.S.); constanza.reyesv@alumnos.uv.cl (C.R.); evdmunoz@gmail.com (E.M.)

[2] Departamento de Ciencias Básicas, Campus Fernando May, Universidad del Bío-Bío, Avda. Andrés Bello 720, Casilla 447, Chillán 3780000, Chile; ewerner@ubiobio.cl

[3] Escuela de Agronomía, Pontificia Universidad Católica de Valparaíso, San Francisco s/n La Palma,
Quillota 2260000, Chile; ximena.besoain@pucv.cl

[4] Center of Interdisciplinary Biomedical and Engineering Research for Health (MEDING), Escuela de
Obstetricia y Puericultura, Facultad de Medicina, Universidad de Valparaíso, Angamos 655,
Reñaca 2520000, Chile; ivan.montenegro@uv.cl

[5] Laboratorio de Pruebas Biológicas, Departamento de Química, Universidad Técnica Federico Santa María, Av. España N1680, Valparaíso 2340000, Chile

* Correspondence: alejandro.madrid@upla.cl (A.M.); katy.diaz@usm.cl (K.D.); Tel.: +56-032-250-0526 (A.M.)

S1. Benzylidene-cycloalkanones (**B**-**I**) NMR data.

(3S)-2-benzylidene-3-isopropenyl-6-methylcyclohexanone (**B**): ^{1}H NMR (400 MHz, $CDCl_3$): δ 7.47 (s, 1H, H-1′); 7.36 (m, 5H, H-3′, H-4′, H-5′, H-6′ and H-7′); 4.76 (s, 1H, H-9β); 4.74 (s, 1H, H-9α); 3.21 (s, 1H, H-3); 2.19 (m, 1H, H-6); 2.04 (m, 1H, H-4b); 1.96 (m, 1H, H-4a); 1.78-1.74 (m, 1H, H-5b); 1.61 (s, 3H, H-8); 1.55-1.46 (m, 1H, H-5a); 1.26 (s, 3H, H-10). ^{13}C NMR (100 MHz, $CDCl_3$): δ 199.7 (C-1); 143.7 (C-2); 141.2 (C-7); 136.9 (C-1′); 136.1 (C-2′); 128.5 (C-3′ and C-7′); 128.4 (C-4′ and C-6′); 126.7 (C-5′); 115.8 (C-9); 47.0 (C-3); 42.5 (C-6); 30.0 (C-5); 29.5 (C-4); 21.0 (C-8); 16.9 (C-10).

(3S)-2-(3-hydroxybenzylidene)-3-isopropenyl-6-methylcyclohexanone (**C**): ^{1}H NMR (400 MHz, $CDCl_3$): δ 7.41 (s, 1H, H-1′); 7.26 (s, 1H, H-3′); 7.21 (m, 1H, H-6′); 6.86 (m, 1H, H-7′); 6.81 (m, 1H, H-5′); 4.99 (s, 1H, H-9β); 4.65 (s, 1H, H-9α); 3.66 (s, 1H, H-3); 2.35 (m, 1H, H-6); 2.02 (m, 1H, H-4b); 1.86-1.77 (m, 1H, H-4a); 1.64 (m, 1H, H-5b); 1.86 (s, 3H, H-8); 1.33-1.25 (m, 1H, H-5a); 1.17 (d, J = 6.6 Hz; 3H, H-10). ^{13}C NMR (100 MHz, $CDCl_3$): δ 206.4 (C-1); 155.7 (C-4′); 145.9 (C-2); 141.6 (C-7); 137.0 (C-1′); 134.9 (C-2′); 129.5 (C-6′); 121.7 (C-7′); 116.4 (C-5′); 115.7 (C-9); 115.1 (C-3′); 45.8 (C-3); 45.5 (C-6); 28.1 (C-5); 27.0 (C-4); 21.5 (C-8); 16.0 (C-10).

(3S)-2-(4-hydroxybenzylidene)-3-isopropenyl-6-methylcyclohexanone (**D**): ^{1}H NMR (400 MHz, $CDCl_3$): ^{1}H NMR (400 MHz, $CDCl_3$): δ 7.48 (s, 1H, H-1′); 7.26 (m, 2H, H-3′ and H-7′); 6.90 (m, 2H, H-4′ and H-6′); 5.01 (s, 1H, H-9$_{\beta}$); 4.71 (s, 1H, H-9$_{\alpha}$); 3.65 (s, 1H, H-3); 2.33 (m, 1H, H-6); 2.07-1.81 (m, 2H, H-4); 1.86 (s, 3H, H-8); 1.45-1.28 (m, 2H, H-5); 1.25 (s, 3H, H-10). ^{13}C NMR (100 MHz, $CDCl_3$): δ 205.2 (C-1); 159.2 (C-5′); 143.7 (C-2); 141.2 (C-7); 136.8 (C-1′); 132.0 (C-3′ and C-7′); 128.9 (C-2′); 115.9 (C-4′ and C-6′); 115.7 (C-9); 46.9 (C-3); 31.9 (C-6); 30.0 (C-5); 29.5 (C-4); 21.0 (C-8); 16.1 (C-10).

(3S)-3-isopropenyl-2-(3-methoxybenzylidene)-6-methylcyclohexanone (**E**): ^{1}H NMR (400 MHz, $CDCl_3$): δ 7.60 (s, 1H, H-1′); 7.27 (m, 1H, H-6′); 6.96 (m, 1H, H-3′); 6.93 (m, 1H, H-7′); 6.89 (m, 1H, H-5′); 5.01 (s, 1H, H-9$_{\beta}$); 4.71 (s, 1H, H-9$_{\alpha}$); 3.79 (s, 3H, OCH_3); 3.66 (s, 1H, H-3); 2.38-1.82 (m, 3H, H-4 and H-6); 1.86 (s, 3H, H-8); 1.46-1.28 (m, 2H, H-5); 1.25 (s, 3H, H-10). ^{13}C NMR (100 MHz, $CDCl_3$): δ 206.4 (C-1); 159.9 (C-4′); 144.9 (C-2); 140.2 (C-7); 137.3 (C-1′); 136.3 (C-2′); 129.5 (C-6′); 122.4 (C-7′); 115.6 (C-9); 114.3 (C-5′); 109.7 (C-3′); 55.2 ($O\underline{C}H_3$); 45.8 (C-3); 44.9 (C-6); 29.7 (C-5); 29.7 (C-4); 21.5 (C-8); 15.9 (C-10).

(3S)-3-isopropenyl-2-(4-methoxybenzylidene)-6-methylcyclohexanone (**F**): ^{1}H NMR (400 MHz, $CDCl_3$): ^{1}H NMR (400 MHz, $CDCl_3$): δ 7.60 (s, 1H, H-1′); 7.26 (m, 2H, H-3′ and H-7′); 6.90 (m, 2H, H-4′ and H-6′); 5.01 (s, 1H, H-9$_{\beta}$); 4.71 (s, 1H, H-9$_{\alpha}$); 3.78 (s, 3H, OCH_3); 3.65 (s, 1H, H-3); 2.03 (m, 1H, H-6); 1.91-1.81 (m, 2H, H-4); 1.87 (s, 3H, H-8); 1.45-1.28 (m, 2H, H-5); 1.25 (s, 3H, H-10). ^{13}C NMR (100 MHz, $CDCl_3$): δ 203.2 (C-1); 159.4 (C-5′); 145.8 (C-2); 139.1 (C-7); 136.5 (C-1′); 129.3 (C-3′ and C-7′); 122.6 (C-2′); 115.1 (C-9); 113.9 (C-4′ and C-6′); 55.3 ($O\underline{C}H_3$); 44.9 (C-3); 31.9 (C-6); 29.7 (C-5); 29.3 (C-4); 21.5 (C-8); 14.1 (C-10).

(3S)-3-isopropenyl-2-(2-methoxybenzylidene)-6-methylcyclohexanone (**G**): ^{1}H NMR (400 MHz, $CDCl_3$): δ 7.44 (s, 1H, H-1′); 7.28 (m, 1H, H-7′); 7.21 (d, *J* = 7.2 Hz, 1H, H-4′); 6.88 (m, 2H, H-5′ and H-6′); 5.00 (s, 1H, H-9$_{\beta}$); 4.67 (s, 1H, H-9$_{\alpha}$); 3.82 (s, 3H, OCH_3); 3.55 (s, 1H, H-3); 2.36 (m, 1H, H-6); 2.10-1.79 (m, 2H, H-4); 1.87-1.62 (m, 2H, H-5); 1.82 (s, 3H, H-8); 1.17 (d, *J* = 3.5 Hz, 3H, H-10). ^{13}C NMR (100 MHz, $CDCl_3$): δ 205.88 (C-1); 158.1 (C-3′); 146.4 (C-7); 130.0 (C-1′); 129.9 (C-5′); 129.4 (C-7′); 124.7 (C-2′); 120.0 (C-6′); 114.9 (C-9); 110.3 (C-4′); 55.4 ($O\underline{C}H_3$); 46.0 (C-3); 45.4 (C-6); 28.3 (C-5); 27.0 (C-4); 21.5 (C-8); 15.9 (C-10).

(3S)-2-(3,4-dimethoxybenzylidene)-3-isopropenyl-6-methylcyclohexanone (**H**): ^{1}H NMR (400 MHz, $CDCl_3$): ^{1}H NMR (400 MHz, $CDCl_3$): δ 7.67 (s, 1H, H-1′); 7.26 (m, 1H, H-3′); 7.00 (m, 1H, H-7′); 6.87

(m, 1H, H-6′); 5.02 (s, 1H, H-9$_β$); 4.75 (s, 1H, H-9$_α$); 3.90 (s, 3H, OCH_3); 3.85 (s, 3H, OCH_3); 3.66 (s, 1H, H-3); 2.01 (m, 1H, H-6); 1.92-1.28 (m, 4H, H-4 and H-5); 1.89 (s, 3H, H-8); 1.25 (m, 3H, H-10). ^{13}C NMR (100 MHz, $CDCl_3$): δ 203.3 (C-1); 150.1 (C-4′); 148.6 (C-5′); 145.5 (C-2); 140.1 (C-7); 134.7 (C-1′); 128.0 (C-2′); 123.4 (C-7′); 114.9 (C-9); 113.0 (C-6′); 110.9 (C-3′); 55.9 (O<u>C</u>H_3); 55.7 (O<u>C</u>H_3); 44.9 (C-3); 32.5 (C-6); 29.7 (C-5); 29.3 (C-4); 21.5 (C-8); 14.1 (C-10). 300.1725).

(3S)-2-(1,3-benzodioxol-5-ylmethylene)-3-isopropenyl-6-methylcyclohexanone (**I**): ^{1}H NMR (400 MHz, $CDCl_3$):): δ 7.58 (s, 1H, H-1′); 6.91 (d, *J* = 8 Hz, 1H, H-7′); 6.85 (m, 1H, H-6′); 6.79 (s, 1H, H-3′); 5.99 (s, 3H, OCH_2O); 4.98 (s, 1H, H-9$_β$); 4.70 (s, 1H, H-9$_α$); 3.66 (s, 1H, H-3); 2.03 (m, 1H, H-6); 1.91-1.28 (m, 4H, H-4 and H-5); 1.89 (s, 3H, H-8); 1.25 (m, 3H, H-10). ^{13}C NMR (100 MHz, $CDCl_3$): δ 203.3 (C-1); 148.4 (C-5′); 147.7 (C-4′); 145.4 (C-2); 139.5 (C-7); 136.6 (C-1′); 129.3 (C-2′); 125.0 (C-7′); 115.4 (C-9); 109.7 (C-6′); 108.4 (C-3′); 101.4 (O<u>C</u>H_2O); 44.7 (C-3); 32.5 (C-6); 29.7 (C-5); 29.3 (C-4); 21.4 (C-8); 14.1 (C-10).

S2. Physico-chemical parameters obteined from SwissADME server.

Compound	Lipophilicity Log P	Heavy Atoms	Aromatic Heavy Atoms	Rot. Bond[a]	H-Bond Acc.[b]	H-Bond Don.[c]	MR[d]	TPSA[e] (A2)
**A**	2.51	11	0	1	1	0	47.80	17.07
**B**	4.02	18	6	2	1	0	77.41	17.07
**C**	3.61	19	6	2	2	1	79.43	37.30
**D**	3.60	19	6	2	2	1	79.43	37.30
**E**	4.01	20	6	3	2	0	83.90	26.30
**F**	4.01	20	6	3	2	0	83.90	26.30
**G**	3.98	20	6	3	2	0	83.90	26.30
**H**	3.96	22	6	4	3	1	90.39	35.53
**I**	3.84	21	6	2	3	1	83.47	35.53

[a]Num. rotatable bonds; [b]Num. H-bond acceptors; [c]Num. H-bond donors; [d]MR, molar refractivity; [e]TPSA, topological polar surface area.
